# Service Availability and Readiness for Essential Health Services in Somalia: A Narrative Review of Facility Evidence and Health‐System Priorities

**DOI:** 10.1002/puh2.70444

**Published:** 2026-11-07

**Authors:** Abdikadir Ahmed Hassan, Mohamed Daud Mohamed, Liban Abdullahi Sudi, Yakub Burhan Abdullahi

**Affiliations:** ^1^ Faculty of Health Sciences and Tropical Medicine Somali National University Mogadishu Somalia; ^2^ Center for Health Research and Innovation Somali National University Mogadishu Somalia; ^3^ Mogadishu Institute of Health Mogadishu Somalia; ^4^ Faculty of Medicine and Health Science, Salaam University Mogadishu Somalia

**Keywords:** equity, essential health services, facility readiness, health‐system strengthening, primary health care, service availability, Somalia, universal health coverage

## Abstract

Essential health services are central to universal health coverage; however, nominal service availability does not necessarily indicate that facilities are ready to deliver care safely, continuously and equitably. In Somalia, evidence on service availability and readiness remains dispersed across facility assessments, policy documents, programme reports and peer‐reviewed studies, limiting its use for planning and equitable resource allocation. This narrative review synthesizes Somalia‐specific evidence on service availability, facility readiness, readiness‐related delivery constraints and health‐system priorities for strengthening essential health services. Evidence was classified as direct facility‐readiness measures, readiness‐related delivery‐system indicators or coverage, programme‐performance and outcome indicators to distinguish structural readiness from indirect signals of delivery‐system performance. Available evidence suggests important readiness gaps across emergency and critical care, surgical services, maternal and newborn health, immunization, nutrition, communicable diseases and selected non‐communicable disease services. These gaps are reinforced by donor‐dependent and geographically uneven financing, fragile supply chains, workforce shortages and maldistribution, weak referral and emergency transport systems, fragmented public, private, NGO and humanitarian service delivery, and limited routine use of facility‐assessment data. Rural, displaced, nomadic, pastoralist and conflict‐affected populations experience substantial access barriers. Institutionalizing readiness monitoring and linking facility‐assessment evidence to planning, budgeting, supervision, supply allocation, donor coordination and accountability could help Somalia move from nominal service availability toward effective, continuous and equitable essential health‐service coverage.

## Graphical abstract

This graphical abstract summarizes the review's central message: In Somalia, the existence of essential health services does not necessarily mean that facilities are ready to deliver safe, continuous and equitable care. Facility‐readiness evidence shows important gaps in emergency and critical care, surgical capacity, immunization, infection prevention and control, workforce availability, supply chains, referral systems, financing and routine data use. These gaps are not isolated facility‐level shortages; they reflect wider health‐system bottlenecks that affect service delivery, particularly for rural, displaced, nomadic, pastoralist and conflict‐affected populations. Strengthening essential health services therefore requires institutionalized readiness monitoring, stronger primary health care platforms, reliable supply chains, workforce planning and retention, functional referral networks and systematic use of facility‐assessment evidence for planning, budgeting, supervision, donor coordination and accountability.

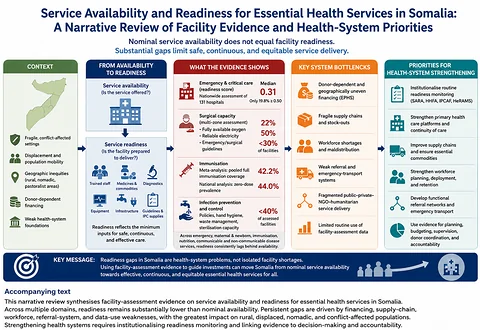

## 1 Introduction

Essential health services are at the core of universal health coverage and primary health care (PHC) efforts, linking access to quality care with broader objectives for population health and health equity [1]. In the SDG era, service coverage and financial risk protection are framed as inseparable from the availability of quality essential medicines, vaccines and health care services [2]. Facility assessment approaches, such as the Service Availability and Readiness Assessment (SARA), operationalize this agenda by measuring both service availability and the readiness of facilities to deliver interventions using standardized tracer indicators [3].

Somalia's health system recovery has occurred in a context shaped by conflict‐related disruption and population displacement, with long‐term effects on the organization, regulation and workforce development [4]. Health system analyses of Somalia have identified rebuilding priorities in governance and management, health financing, private sector engagement and human resources for health [5]. Available evidence also suggests that health service delivery is concentrated in major towns, leaving rural communities with limited access to affordable care [6].

Nominal service availability does not necessarily indicate real service delivery capacity. A facility may be counted as providing a service but still lack the inputs required for safe, continuous and effective care [3]. The SARA framework highlights how readiness gaps can span laboratories and essential medicines and commodities, undermining the ability to deliver interventions expected under essential health services [3]. Somalia‐specific emergency and critical care evidence similarly reports low readiness, with barriers including the absence of staff availability around the clock, lack of equipment and user fees [7].

In Somalia, facility assessment evidence is important because readiness constraints are described across the health system, including workforce and referral weaknesses that affect the delivery of maternal and newborn care [8]. A Somalia emergency and essential surgical care assessment across all three administrative zones (Somaliland, Puntland and South/Central Somalia) reported that only 22% of assessed facilities had fully available oxygen access and 50% had fully available electricity, whereas management guidelines for emergency and surgical care were also limited [9]. Where service delivery is shaped by under‐resourcing, facility readiness assessment findings can support the prioritization of investments for priority services, workforce development and broader health system strengthening within the EPHS policy process [10].

Evidence on service availability and readiness in Somalia remains dispersed across facility assessments, programme reports, policy documents and peer‐reviewed studies, limiting its usability for strategic planning and equitable resource allocation in the country [11]. Recent Somalia‐specific analyses emphasize that EPHS financing is donor‐dominated and varies by region, underscoring that readiness strengthening should account for geographic inequities across stable and conflict‐affected areas [12]. This narrative review synthesizes facility assessment evidence on service availability and readiness for essential health services in Somalia and identifies priorities for strengthening the health system.

## 2 Methods

### 2.1 Review Design

This narrative review synthesized Somalia‐specific evidence on service availability, facility readiness, readiness‐related delivery constraints and health‐system priorities for essential health services. A narrative approach was selected because the available evidence is methodologically heterogeneous, spanning facility assessments, health‐system analyses, programme evaluations, qualitative studies, policy documents and official reports. The review was designed to clarify the distinction between nominal service availability and facility readiness and to identify how readiness evidence can inform health‐system strengthening in Somalia.

### 2.2 Search Strategy and Information Sources

We searched peer‐reviewed and grey‐literature sources relevant to facility readiness and essential health‐service delivery in Somalia. Peer‐reviewed literature was identified through PubMed/MEDLINE, Google Scholar and cross‐reference searching of relevant articles. Grey‐literature sources included World Health Organization (WHO) resources, Somali Ministry of Health and EPHS‐related documents, World Bank and UNICEF materials, Health Cluster and humanitarian health‐system sources and official facility‐assessment or health‐sector reports where available. Searches were supplemented by screening the reference lists of included articles and reports.

Search terms combined Somalia‐specific terms with service‐readiness and health‐system terms. Indicative search terms included ‘Somalia’ OR ‘Somaliland’ OR ‘Puntland’; ‘service availability’; ‘facility readiness’; ‘health facility assessment’; ‘SARA’; ‘HeRAMS’; ‘HHFA’; ‘IPCAF’; ‘primary health care’; ‘essential health services’; ‘EPHS’; ‘emergency care’; ‘surgical capacity’; ‘maternal and newborn health’; ‘immunization’; ‘nutrition’; ‘communicable diseases’; ‘non‐communicable diseases’; ‘health workforce’; ‘supply chain’; ‘referral’ and ‘universal health coverage’. The searches covered the literature available up to September 2026.

### 2.3 Eligibility Criteria

Sources were eligible if they reported evidence relevant to service availability, facility readiness, health‐service delivery capacity or health‐system bottlenecks affecting essential health services in Somalia. We included peer‐reviewed studies, facility assessments, systematic or narrative reviews, policy analyses, qualitative studies, official reports and health‐sector documents if they contained Somalia‐specific evidence or directly relevant methodological or conceptual guidance. Sources were excluded if they did not address Somalia, reported only on Ethiopia's Somali Region or Somali populations outside Somalia without clear relevance to Somalia's health system, lacked relevance to facility readiness or essential service delivery or could not be verified through a DOI, journal page or official institutional source.

### 2.4 Study Selection and Data Extraction

Potentially relevant sources were screened by title, abstract, executive summary and full text where available. Information was extracted on publication year, setting, administrative zone or region, facility type, study design, sample or assessment frame, tool used, service area, readiness domains assessed, key findings and evidence limitations. For facility assessments, the extracted domains included workforce, training, guidelines, medicines, vaccines, diagnostics, equipment, oxygen, electricity, water, sanitation, infection prevention and control (IPC), referral capacity, transport, communication and data systems. For health‐system and policy sources, the extracted information focused on financing, governance, donor dependence, private‐sector engagement, supply chains, workforce planning, referral systems, data use and accountability.

### 2.5 Evidence Classification

To address differences in measurement and interpretation, evidence was classified into three categories. First, direct facility‐readiness measures included facility‐level assessments of structural inputs such as staff, medicines, diagnostics, equipment, infection‐prevention supplies, oxygen, electricity, guidelines, infrastructure and service‐specific readiness scores. Second, readiness‐related delivery‐system indicators included evidence on referral capacity, outreach delivery, cold‐chain continuity, supply reliability, workforce distribution, ambulance availability, blood availability and service continuity. Third, coverage, utilization, programme‐performance or outcome indicators included institutional delivery, immunization coverage, zero‐dose prevalence, nutrition programme recovery, outbreak burden and mortality‐related indicators. These indicators were not interpreted as direct readiness measures but were included when they helped identify delivery‐system constraints relevant to effective essential health‐service coverage.

### 2.6 Assessment of Evidence Limitations

Because the included sources varied in design, tool, population and purpose, we did not conduct a formal meta‐analysis. Instead, we assessed each source narratively according to its relevance to facility readiness, geographic scope, facility or population coverage, sampling frame, tool or indicator definition, representativeness and potential limitations. Particular attention was given to whether findings were based on national, multi‐zone, regional, facility‐based, qualitative or programme‐specific evidence; whether private, NGO‐supported, humanitarian, rural, insecure, displaced, nomadic or pastoralist populations were represented; and whether indicators measured direct readiness or indirect readiness‐related constraints. Where evidence was indirect, service‐specific or geographically limited, this was stated explicitly.

### 2.7 Synthesis Approach

Findings were synthesized thematically around the review's conceptual framing: service availability, facility readiness, effective access and health‐system strengthening. The synthesis first examined evidence on geographic service availability and general readiness, then summarized readiness‐related evidence across priority service areas, equity gaps in effective access, health‐system bottlenecks and policy priorities. The synthesis emphasized Somalia‐specific evidence while using international facility‐readiness concepts and tools to interpret the findings. The goal was not to estimate pooled readiness levels but to identify recurring readiness gaps, clarify the strength and limitations of the evidence and derive practical priorities for health‐system strengthening.

## 3 Conceptual Framing: From Service Availability to Facility Readiness

A health facility that offers a service may nonetheless lack the inputs required to deliver it safely, a distinction that lies at the heart of facility assessment frameworks and carries direct implications for health system planning (Figure 1) [3, 13]. The SARA formalizes this distinction as a standardized, WHO‐endorsed framework that measures two complementary dimensions: service availability, defined as the reported presence and geographic distribution of services or delivery points within a defined area, and service readiness, defined as the degree to which a facility possesses the minimum inputs needed to deliver those services reliably [3, 13]. Related tools, including the Service Provision Assessment (SPA) and the Health Resources and Services Availability Monitoring System (HeRAMS), also generate facility‐ and service‐level information for health‐system monitoring, although their purposes and contexts of use differ [14, 15].

**FIGURE 1:**
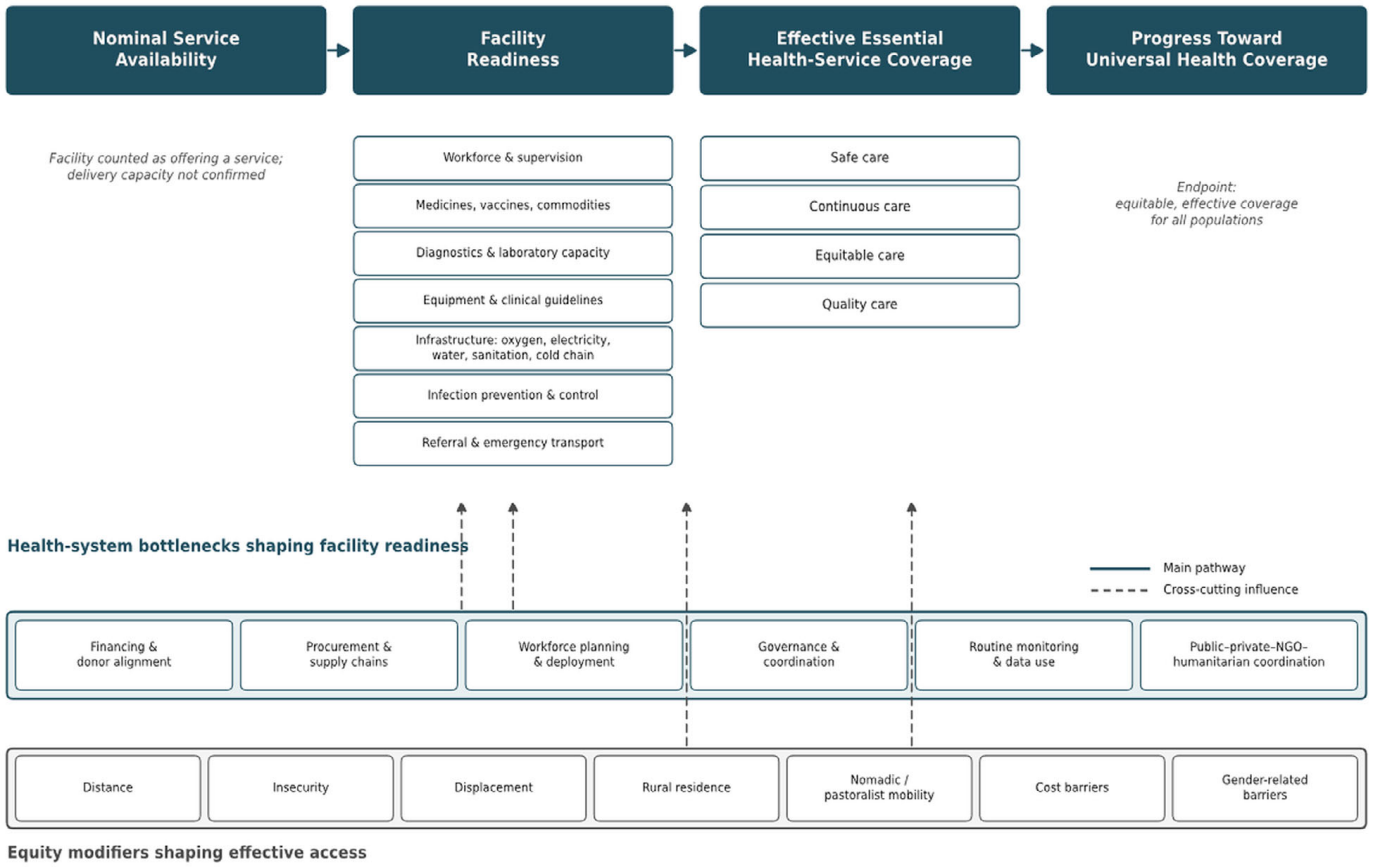
Conceptual framework linking nominal service availability, facility readiness, effective essential health‐service coverage and progress toward universal health coverage in Somalia. Nominal service availability reflects the reported presence of services or facilities, whereas facility readiness reflects the inputs required to deliver care safely and continuously. Health‐system bottlenecks, including financing, workforce, supply chains, infrastructure, data systems, governance and referral capacity, shape readiness. Equity modifiers, including displacement, rural residence, nomadic and pastoralist mobility, gender, disability, affordability and insecurity, influence whether available and ready services translate into effective access.

SARA operationalizes service readiness through tracer items that commonly cover trained staff, clinical guidelines, essential medicines and vaccines, diagnostics, basic equipment, enabling infrastructure and IPC supplies [3, 13]. Readiness is quantified as the mean proportion of tracer items present across these domains, producing a facility‐level index that serves as a measurable proxy for service delivery capacity rather than direct evidence of clinical quality or patient outcomes [3, 14, 16]. The interpretation of readiness scores is study‐specific and should be contextualized against the assessment tool, facility type and service domain [14, 16]. Available evidence from multi‐country SPA and SARA applications suggests that deficiencies in essential medicines and diagnostic capacity are among the most frequently identified bottlenecks and that tracer‐based assessments can detect input gaps that aggregate availability counts may not [16].

In this review, the conceptual framework was operationalized by distinguishing three forms of evidence. Direct facility‐readiness measures were defined as facility‐level indicators of structural inputs, including workforce, medicines, diagnostics, equipment, oxygen, electricity, water, sanitation, infection‐prevention supplies, clinical guidelines and service‐specific readiness scores. Readiness‐related delivery‐system indicators included referral capacity, outreach delivery, cold‐chain continuity, commodity reliability, ambulance availability, blood availability, workforce distribution and service continuity. Coverage, utilization, programme‐performance and outcome indicators, including institutional delivery, immunization coverage, zero‐dose prevalence, nutrition programme recovery, outbreak burden and mortality‐related indicators, were not interpreted as direct readiness measures. They were included only when they helped identify delivery‐system constraints relevant to effective essential health‐service coverage. This distinction guided the synthesis, the interpretation of Table 1 and the policy priorities proposed below.

**TABLE 1:** Direct readiness, readiness‐related and coverage indicators by priority service area in Somalia.

Service area	Evidence type	Design, tool, sample and year	Main Somalia‐specific finding	Interpretation and key evidence gap
Emergency and critical care	Direct facility‐readiness measure	Cross‐sectional nationwide facility assessment using the WHO Hospital Emergency Unit tool; 131 hospitals; 2025	Median readiness score was 0.31; 19.8% of hospitals exceeded the 0.50 readiness threshold [7]	Indicates limited readiness for triage, resuscitation and stabilization in assessed hospitals. No repeated rounds, limited linkage to patient outcomes and limited PHC‐level emergency‐care evidence
Surgical care	Direct facility‐readiness measure	Cross‐sectional multi‐zone survey across Somaliland, Puntland and South‐Central Somalia; 2014; complemented by national surgical, obstetric and anaesthesia workforce analysis	Fully available oxygen was reported in 22% of facilities, reliable electricity in 50%, and emergency/surgical guidelines in fewer than 30%; the specialist workforce was concentrated in Banadir [9, 23]	Indicates constrained surgical, anaesthesia and emergency obstetric capacity. The 2014 facility survey has not been repeated, and private, NGO and perioperative outcome data remain limited
Maternal and newborn health	Readiness‐related delivery‐system indicator	Narrative review of MNH system challenges, complemented by qualitative evidence from pastoralist‐serving clinics in Mudug	Workforce, commodity, blood, ambulance and emergency obstetric referral constraints have been reported [8, 25]	Suggests readiness constraints for emergency obstetric and newborn care, particularly in rural and pastoralist‐serving settings. Facility‐level EmONC signal‐function assessments and outcome‐linked readiness data remain limited
Immunization	Coverage/programme‐performance indicator	Systematic review and meta‐analysis of 11 studies; SHDS 2020 secondary analysis; qualitative evidence on mobile and displaced populations	Pooled full immunization coverage was 42.2%; four‐vaccine completion was 16.1%; national zero‐dose prevalence was 44.0%, with marked regional disparities [27, 28, 29]	These are not direct facility‐readiness measures. They indicate delivery‐system constraints in outreach, microplanning, cold‐chain continuity and follow‐up, especially for mobile, displaced and insecure populations
Nutrition	Coverage/programme‐performance indicator	Systematic review and meta‐analysis of integrated management of acute malnutrition programmes in Somalia	Pooled outpatient therapeutic programme recovery was 95.4%; stabilization‐centre recovery was 80.8% [30]	Reflects programme performance rather than system‐wide facility readiness. Facility‐level data on therapeutic commodities, staff competence, inpatient SAM care, relapse and mortality remain limited
Communicable diseases	System‐pressure indicator	Narrative review of communicable‐disease control, surveillance and outbreak response	Recurrent measles, cholera and polio outbreaks have been reported alongside constrained surveillance and diagnostic capacity [31]	Outbreak burden should be interpreted as pressure on surveillance, diagnostics, IPC and continuity of care, not as direct facility‐readiness evidence. Facility‐level diagnostic and outbreak‐response readiness evidence remains limited
Non‐communicable diseases	Direct facility‐readiness measure	Cross‐sectional assessment of seven hospitals in two Somaliland regions	Workforce shortages, scarce advanced diagnostics and inconsistent essential medicine and guideline availability were reported for hypertension, diabetes and related NCD conditions [26]	Indicates constrained readiness for NCD detection, treatment initiation and continuity in assessed Somaliland hospitals. Comparable PHC‐level and federal member state evidence remains limited

*Note:* Evidence was classified as direct facility‐readiness measures, readiness‐related delivery‐system indicators, coverage/programme‐performance indicators or system‐pressure indicators. Coverage, programme‐performance, outbreak and access indicators were not interpreted as direct facility‐readiness measures; they were included only when they identified delivery‐system constraints relevant to effective essential health‐service coverage.

Abbreviations: EmONC, emergency obstetric and newborn care; IMAM, integrated management of acute malnutrition; IPC, infection prevention and control; MNH, maternal and newborn health; NCD, non‐communicable disease; PHC, primary health care; SAM, severe acute malnutrition; SHDS, Somali Health and Demographic Survey; WHO, World Health Organization.

## 4 Facility Assessment Evidence on Essential Health Services in Somalia

### 4.1 Service Availability and Geographic Coverage

Available evidence suggests that access to facility‐based essential services in Somalia is uneven and varies markedly by region [6, 12, 17]. Analysis of the 2020 Somali Health and Demographic Survey (SHDS) reported that 22.3% of women delivered in a health facility nationally, ranging from 58% in Woqooyi Galbeed to 2% in Bakool, with rural or nomadic residence and distance independently associated with lower odds of institutional delivery [17]. An equity analysis of the 2021–2026 Essential Package of Health Services reported donor‐dominated funding and per‐capita allocation two to three times higher in Somaliland, Puntland and Banadir than in Southwest and Hirshabelle, with funding inversely correlated with under‐five mortality [12].

These access gaps are particularly important for displaced, rural, nomadic and pastoralist populations [18, 19, 20]. Among internally displaced persons in Mogadishu and Baidoa, residence more than 2 km from a facility was associated with higher odds of foregoing care [18]. In Hargeisa, 73.5% of displaced households fell into the lowest utilization tier, with affordability and accessibility reported as dominant factors [19]. Mixed‐methods evidence from pastoralists across Galmudug, Puntland and Somaliland reported distance and cost as major barriers to care [20]. These findings are not direct facility‐readiness measures, but they show how geographic access, financing, mobility and affordability can limit effective service coverage even where services are nominally available.

### 4.2 General Facility Readiness

Direct facility‐readiness assessments indicate that nominal service provision should not be equated with delivery capacity [7, 9, 21, 22]. A nationwide assessment of 131 hospitals using the WHO Hospital Emergency Unit tool reported a median emergency and critical care readiness score of 0.31, with 19.8% of hospitals reaching the study‐defined threshold of 0.50 [7]. A multi‐zone surgical capacity survey across Somaliland, Puntland and South‐Central Somalia found that 22% of facilities had fully available oxygen, 50% had reliable electricity, and fewer than 30% had management guidelines for emergency or surgical care [9]. IPC assessments also show important readiness gaps. A secondary analysis of the 2024 Harmonized Health Facility Assessment of 1219 facilities across six federal member states reported standard‐precaution policies in 24% of facilities and waste‐management policies in 18% [21]. An IPCAF assessment of 307 facilities returned a mean score of 136 out of 800 [22]. Workforce evidence further indicates low availability and maldistribution of skilled personnel: A national surgical workforce assessment reported 1.2 surgical, 0.4 obstetric and 0.2 anaesthesia specialists per 100,000 population [23], whereas a health‐system synthesis reported 4.3 core health workers and 0.4 physicians per 10,000 population, with rural maldistribution and clinician emigration [24]. Together, these direct readiness and workforce findings identify structural constraints in staffing, infrastructure, IPC, equipment and service organization [24]. Qualitative work in pastoralist‐serving maternal and child health clinics in Mudug reported missing supplies, staff, ambulances and blood for transfusion, undermining referral capacity where services were offered [25].

### 4.3 Readiness for Priority Service Areas

Readiness evidence is strongest for emergency and critical care, surgical care, maternal and newborn health (MNH), immunization, nutrition and selected non‐communicable disease (NCD) services, whereas comparable facility‐readiness evidence for other priority areas remains limited [7, 8, 26]. Table 1 summarizes direct facility‐readiness measures, readiness‐related delivery‐system indicators and coverage or programme‐performance indicators across seven priority essential health‐service areas.

Emergency, critical and surgical care are supported by direct facility‐readiness evidence from the nationwide hospital assessment and the multi‐zone surgical capacity survey [7, 9]. MNH evidence is more mixed, combining health‐system reviews and qualitative evidence on workforce, commodities, blood availability, ambulances and referral constraints [8, 25]. Immunization evidence is mainly coverage‐ and delivery‐system‐related rather than direct facility readiness: a systematic review reported low pooled full immunization coverage, whereas SHDS‐based analysis reported high zero‐dose prevalence and regional disparities [27, 28, 29]. Nutrition evidence similarly reflects programme performance rather than full facility readiness, with integrated management of acute malnutrition (IMAM) evidence suggesting differences between outpatient and stabilization‐centre performance [30]. Communicable‐disease evidence indicates surveillance and continuity‐of‐care pressures, but outbreak burden should not be interpreted as a direct readiness measure [31]. For NCDs, available facility evidence remains limited to selected Somaliland hospitals and should not be generalized nationally [26].

### 4.4 Equity Gaps in Effective Service Access

The available evidence indicates that readiness gaps intersect with inequities in geography, financing, displacement, mobility and workforce distribution [12, 18, 19, 20, 23]. EPHS financing inequities suggest that resource allocation does not consistently match population needs, particularly in conflict‐affected regions [12]. IDP evidence from Mogadishu, Baidoa and Hargeisa shows that distance, affordability and accessibility can reduce utilization among displaced populations [18, 19]. Pastoralist evidence from Galmudug, Puntland and Somaliland similarly identifies distance and cost as major barriers to care [20]. These equity findings should be interpreted as access and delivery‐system evidence rather than direct facility‐readiness measurement. However, they are important because facility readiness cannot translate into effective coverage if services remain physically inaccessible, unaffordable, weakly referred or concentrated in better served regions. Workforce maldistribution may further compound these inequities, particularly where specialist and referral capacity are concentrated in urban centres [23]. Future readiness monitoring should therefore include equity‐sensitive indicators that capture facility location, ownership, catchment populations, displacement status, nomadic and pastoralist mobility, disability inclusion, gender‐related access barriers and referral functionality.

## 5 Health‐System Bottlenecks Behind Readiness Gaps

The facility‐readiness deficits described above should be interpreted as interlocking system‐level bottlenecks rather than isolated facility shortages. Financing is a central constraint. Available evidence suggests that Essential Package of Health Services financing remains donor‐dominated and geographically uneven, with higher per‐capita allocation in Somaliland, Puntland and Banadir than in Southwest and Hirshabelle [12]. Project‐based financing and fragmented partner priorities may therefore reinforce unequal readiness across regions and service platforms, particularly where conflict and displacement increase health need but reduce implementation capacity [10, 12, 24].

Supply‐chain weaknesses are another recurrent bottleneck. Gaps in infection‐prevention inputs, sterilizing equipment, medicines, oxygen, blood, ambulances and other commodities suggest weaknesses in forecasting, procurement, storage, distribution and last‐mile delivery [7, 21, 22, 25]. These gaps matter because facility readiness depends not only on the existence of buildings or service labels but also on the continuous availability of essential inputs. When supply chains are unreliable, immunization, maternal and newborn care, nutrition, emergency care, surgery, infection prevention and NCD services may be nominally available but operationally fragile.

Workforce constraints further limit readiness. Somalia's low density of core health workers and physicians, clinician emigration, rural maldistribution and concentration of specialist surgical, obstetric and anaesthesia personnel in Banadir reduce the ability of facilities outside major urban centres to provide continuous, supervised and specialized care [23, 24]. Multi‐stakeholder consultations on the 2025–2029 National Transformation Plan identified workforce regulation, training‐hub coverage and rural‐deployment incentives as priorities for implementation [32]. These workforce bottlenecks affect not only service coverage but also quality, supervision, referral decisions and continuity of care.

Infrastructure and referral systems also shape readiness. Facility assessments and service‐specific studies indicate persistent constraints in oxygen, electricity, emergency‐care capacity, ambulances, blood availability, communication and referral pathways [7, 8, 9, 25]. These are not background conditions; they are core readiness inputs. Emergency obstetric, newborn, surgical and critical care services depend on functioning referral chains, reliable communication between facilities, transport capacity and receiving facilities that are prepared to manage referred patients [8, 25]. Weaknesses at any point in this chain can reduce the value of nominal service availability.

Service delivery remains fragmented across public, private, NGO‐supported and humanitarian providers. Health‐system strengthening analyses have emphasized the need for stronger governance, coordination, financing, human resources and engagement with Somalia's large private health sector [5, 32]. This fragmentation can weaken standard‐setting, reporting, referral coordination, supervision and accountability, especially when humanitarian and routine systems operate through parallel planning and funding mechanisms. Although DHIS2 reporting has expanded and tools such as HHFA, IPCAF, HeRAMS and SARA‐type assessments have been used, routine use of readiness data for budgeting, supply allocation, supervision, donor coordination and accountability remains limited by interoperability, data‐quality and rural‐connectivity constraints [21, 24]. These bottlenecks suggest that strengthening essential health services in Somalia requires moving beyond nominal service availability toward facilities that are staffed, supplied, equipped, supervised, financed, monitored and connected through functioning referral systems.

## 6 Policy Priorities for Health‐System Strengthening

The readiness deficits and system bottlenecks synthesized above suggest a sequenced policy agenda that is feasible for Somalia's federal, donor‐supported and security‐affected health system. The immediate priority should be to institutionalize routine facility readiness monitoring. In the short term, a minimum readiness module could be embedded within DHIS2/HMIS supervision checklists and linked to quarterly district review meetings, whereas HeRAMS could be used more selectively for crisis‐affected or access‐constrained districts where routine reporting is incomplete [15, 21, 24]. This would clarify the added value of each platform: DHIS2 can support routine management, supervision and trend monitoring, whereas HeRAMS can support rapid mapping of service availability and functionality in humanitarian or hard‐to‐reach settings [15, 24]. The Federal Ministry of Health could define national readiness indicators and reporting standards, whereas state ministries and districts could use dashboards to prioritize supervision, supply allocation and facility follow‐up.

PHC platforms should be strengthened as the operational base for the Essential Package of Health Services. In the medium term, PHC consolidation should focus on a realistic package that integrates maternal and newborn care, child health, immunization, nutrition, communicable‐disease services, basic NCD detection, referral and continuity of care [8, 10, 12, 29]. This does not require expanding every service everywhere at once; rather, districts could phase implementation by prioritizing high‐burden catchments, underserved IDP and pastoralist populations and facilities with the largest readiness gaps [12, 18, 19, 20]. This approach could reduce fragmentation between humanitarian and routine service streams while aligning partner support with EPHS priorities.

Supply‐chain strengthening should be pursued alongside PHC consolidation. Short‐ to medium‐term actions include district‐level consumption tracking, pooled procurement under EPHS‐aligned commodity lists, cold‐chain maintenance, oxygen planning, buffer stocks for insecure districts and partner coordination around last‐mile delivery [7, 21, 22, 25]. These actions are more feasible than broad calls to ‘increase funding’ because they link financing to specific commodities, facility gaps and service platforms. Donors, NGOs and government procurement units should use readiness data to identify where medicines, vaccines, diagnostics, IPC supplies, nutrition commodities, oxygen, blood and basic equipment are missing or unreliable.

Workforce policy requires both immediate supervision and longer term investment. In the short term, structured supportive supervision, mentorship and task‐sharing protocols could improve the performance of existing staff. In the longer term, regulated pre‐service training, regional training hubs, rural‐deployment incentives, retention packages and specialist workforce planning are needed to address workforce density gaps, rural maldistribution, clinician emigration and specialist concentration in Banadir [23, 24, 32]. Federal authorities should set standards and accreditation mechanisms, whereas state authorities should lead deployment, supervision and retention strategies adapted to local needs.

Referral, emergency care, communication and transport systems should also be built progressively. District referral protocols, basic inter‐facility communication, ambulance coordination, oxygen referral pathways and blood availability can be prioritized first for maternal, newborn, surgical and critical‐care services [7, 8, 9, 25]. Over time, hub‐and‐spoke referral networks linking PHC facilities, district hospitals and higher level hospitals could improve continuity and reduce delays. Private, NGO‐supported and humanitarian providers should be included in referral mapping and reporting because many patients move across provider types.

Finally, facility‐assessment evidence should be used for planning, budgeting, donor coordination and accountability. Annual EPHS budgeting and partner mapping should be linked to readiness gaps, not only to facility counts or programme targets [10, 12, 24]. In the short term, district readiness reviews can identify practical corrective actions; in the medium term, readiness indicators can guide donor alignment and supervision; and in the longer term, readiness improvement should become a measurable health‐system strengthening objective in Somalia, not an assumed consequence of expanding service availability.

## 7 Evidence Gaps and Future Research

The synthesis highlights several evidence gaps that limit the policy use of facility‐assessment evidence in Somalia. First, nationally representative and repeatable facility‐readiness assessments remain limited. Existing evidence includes important service‐specific and facility‐based assessments, but differences in tools, sampling frames, facility definitions, ownership coverage and service domains constrain comparison across federal member states, facility levels and time periods [7, 9, 21, 22]. Future assessment rounds should use a harmonized core set of readiness indicators that can be repeated over time and linked to EPHS planning, district budgeting, supervision and donor coordination [10, 12, 24].

Second, future research should better distinguish direct readiness, readiness‐related delivery‐system indicators and coverage or outcome indicators. Existing evidence on institutional delivery, zero‐dose prevalence, immunization coverage, nutrition programme recovery and outbreak burden is valuable, but these indicators do not directly measure whether facilities have the staff, commodities, equipment, diagnostics, infrastructure, guidelines and referral systems required for safe and continuous care [17, 27, 28, 29, 30, 31]. Future studies should link facility‐readiness measures with service utilization, referral completion, patient outcomes, financial protection and equity indicators to clarify how readiness affects effective coverage and progress toward universal health coverage.

Third, the evidence base remains uneven across provider types and populations. Somalia's mixed service‐delivery system includes public, private, NGO‐supported, faith‐based and humanitarian providers, but these are not consistently represented in assessment frames [5, 6, 24]. Insecure districts, rural communities, IDPs, nomadic and pastoralist populations, women, children and persons with disabilities also require more explicit attention in readiness monitoring [18, 19, 20, 25]. Equity‐sensitive facility assessment should therefore include catchment characteristics, displacement status, mobility patterns, disability inclusion, gender‐related access barriers, affordability and referral functionality.

Fourth, future research should move beyond structural inputs alone. Facility assessments should be complemented by measures of clinical process quality, provider competence, patient safety, respectful care, continuity of care, referral completion and service functionality. Methods such as observation of care, clinical vignettes, exit interviews, routine outcome linkage and implementation research could help determine whether readiness improvements translate into better care. Costing and cost‐effectiveness studies are also needed to compare readiness‐improvement packages, such as workforce supervision, supply‐chain strengthening, oxygen systems, emergency referral, cold‐chain maintenance and PHC consolidation.

Fifth, facility‐assessment data need stronger integration into routine decision‐making. DHIS2/HMIS, HHFA, IPCAF, HeRAMS and SARA‐type tools should not operate as parallel reporting systems; they should inform supply allocation, supportive supervision, district performance review, donor mapping and accountability [21, 24]. Strengthening Somalia's facility‐assessment evidence base is therefore not only a research priority but also a practical requirement for planning, budgeting, performance monitoring, donor coordination, equity and tracking progress toward universal health coverage.

A further evidence gap concerns epistemic trust and local ownership of facility‐assessment data. In fragile settings, readiness data are more likely to influence policy when they are not only technically valid but also trusted by federal and state authorities, facility managers, frontline workers, communities and implementing partners. Recent work on epistemic justice and knowledge systems emphasizes that evidence systems can marginalize local knowledge when data production, interpretation and decision‐making are externally driven. For Somalia, future readiness monitoring should therefore involve Somali institutions, state health authorities, facility teams, private and humanitarian providers and affected communities in indicator selection, interpretation and use. This would strengthen the legitimacy of readiness evidence and increase its likelihood of being used for planning, budgeting, supervision and accountability [33].

## 8 Limitations

This review has several limitations. First, although a structured search strategy and explicit eligibility criteria were used, the review was narrative rather than systematic. Some relevant grey‐literature reports, partner assessments or unpublished facility data may therefore have been missed, particularly where documents were not indexed, publicly available or easily retrievable. Second, the included evidence was heterogeneous in design, purpose, sampling frame, geography, facility type, ownership and measurement tool. Findings from HHFA, IPCAF, HeRAMS, SARA‐type tools, hospital emergency assessments, qualitative studies, programme reviews and policy analyses are therefore not directly comparable, and readiness scores should be interpreted within the context of each instrument and facility sample.

Third, several included indicators were not direct facility‐readiness measures. Coverage, utilization, programme performance, outbreak and access indicators were included only when they helped identify readiness‐related delivery‐system constraints, but they should not be interpreted as substitutes for facility‐level readiness assessment. Fourth, the representativeness of available evidence is uneven. Private‐sector, NGO‐supported, faith‐based, humanitarian, rural, insecure, displaced, nomadic, pastoralist and disability‐affected populations are not consistently captured in existing facility assessments, limiting conclusions about equity‐sensitive readiness. Fifth, many assessments emphasize structural inputs, such as staff, equipment, guidelines, medicines and infrastructure, while providing limited information on clinical processes, provider competence, patient safety, respectful care, continuity of care, referral completion or patient outcomes.

Finally, this review did not conduct a meta‐analysis or formal risk‐of‐bias scoring across all included sources because of substantial methodological heterogeneity. Causal links between facility readiness, service utilization, equity and health outcomes should therefore be interpreted cautiously. Despite these limitations, the synthesis identifies recurring readiness gaps and health‐system bottlenecks that are relevant for planning, supervision, donor coordination and future facility‐assessment design in Somalia.

## 9 Conclusion

This review shows that Somalia's essential health‐service challenge is not only one of nominal service availability but also of facility readiness. The available evidence suggests that readiness gaps affect multiple service areas, including emergency and critical care, surgical services, MNH, immunization, nutrition, communicable diseases and selected NCD services. However, the evidence base is uneven: Some findings derive from direct facility‐readiness assessments, whereas others reflect readiness‐related delivery constraints, programme performance, access barriers or system pressures.

The synthesis indicates that readiness gaps are shaped by interlocking health‐system bottlenecks, including donor‐dependent and geographically uneven financing, fragile supply chains, workforce shortages and maldistribution, weak referral and emergency transport systems, fragmented service delivery and limited routine use of readiness data. These constraints are most consequential for populations already facing barriers to care, including rural communities, internally displaced people, nomadic and pastoralist populations and conflict‐affected groups.

Strengthening essential health services in Somalia therefore requires moving beyond counting facilities or services toward measuring and improving the inputs and systems that make care deliverable. Facility‐readiness monitoring should be institutionalized as a routine decision‐making tool linked to planning, budgeting, supervision, supply allocation, donor coordination, referral‐system strengthening and accountability. Readiness improvement should be treated as a measurable objective of health‐system strengthening and progress toward universal health coverage, rather than as an assumed consequence of expanding service availability.

## Author Contributions

**Abdikadir Ahmed Hassan** conceptualized the study. **Liibaan Abdulahi Sudi** developed the methodology, conducted the investigation and analysis and prepared the original manuscript. **Mohamed Daud Mohamed** critically reviewed and edited the manuscript. **Yakub Burhan Abdullahi** provided supervision and guidance for the study. All authors have read and approved the final manuscript.

## Funding

The authors have nothing to report.

## Ethics Statement

The authors have nothing to report.

## Consent

The authors have nothing to report.

## Conflicts of Interest

The authors declare no conflicts of interest.

## Data Availability

No new data were generated or analysed in this study. Therefore, data sharing was not applicable to this study.
